# Age of acquisition of 299 words in seven languages: American English, Czech, Gaelic, Lebanese Arabic, Malay, Persian and Western Armenian

**DOI:** 10.1371/journal.pone.0220611

**Published:** 2019-08-08

**Authors:** Magdalena Łuniewska, Zofia Wodniecka, Carol A. Miller, Filip Smolík, Morna Butcher, Vasiliki Chondrogianni, Edith Kouba Hreich, Camille Messarra, Rogayah A. Razak, Jeanine Treffers-Daller, Ngee Thai Yap, Layal Abboud, Ali Talebi, Maribel Gureghian, Laurice Tuller, Ewa Haman

**Affiliations:** 1 University of Warsaw, Faculty of Psychology, Warsaw, Poland; 2 Jagiellonian University, Institute of Psychology, Krakow, Poland; 3 Pennsylvania State University, Department of Communication Sciences and Disorders, University Park, Pennsylvania, United States of America; 4 Czech Academy of Sciences, Institute of Psychology, Prague, Czech Republic; 5 University of Edinburgh, School of Philosophy, Psychology and Language Sciences, Edinburgh, Scotland, United Kingdom; 6 Saint Joseph University of Beirut, Faculty of Medicine, Beirut, Lebanon; 7 Saint Joseph University of Beirut, High Institute of Speech and Language Therapy, Beirut, Lebanon; 8 Universiti Kebangsaan Malaysia, Faculty of Health Science, Kuala Lumpur, Malaysia; 9 University of Reading, Department of English Language and Applied Linguistics, Reading, United Kingdom; 10 Universiti Putra Malaysia, Faculty of Modern Languages and Communication, Serdang, Malaysia; 11 Allameh Tabatabai University, Department of Linguistics and Teaching Persian to Speakers of Other Languages, Teheran, Iran; 12 University of Tours, UMR 1253, iBrain, Tours, France; Centre National de la Recherche Scientifique, FRANCE

## Abstract

We present a new set of subjective Age of Acquisition (AoA) ratings for 299 words (158 nouns, 141 verbs) in seven languages from various language families and cultural settings: American English, Czech, Scottish Gaelic, Lebanese Arabic, Malaysian Malay, Persian, and Western Armenian. The ratings were collected from a total of 173 participants and were highly reliable in each language. We applied the same method of data collection as used in a previous study on 25 languages which allowed us to create a database of fully comparable AoA ratings of 299 words in 32 languages. We found that in the seven languages not included in the previous study, the words are estimated to be acquired at roughly the same age as in the previously reported languages, i.e. mostly between the ages of 1 and 7 years. We also found that the order of word acquisition is moderately to highly correlated across all 32 languages, which extends our previous conclusion that early words are acquired in similar order across a wide range of languages and cultures.

## Introduction

### Age of acquisition effect

Age of acquisition (AoA), i.e. an estimation of the age at which a word is acquired, is an important factor in research on word processing. A growing number of studies have shown AoA to affect performance in various psycholinguistic tasks, such as picture naming, word naming or lexical decision [1–3]. In particular, words acquired earlier in life are processed faster and more accurately than words learned later. This phenomenon is called “Age of acquisition effect”. Recently, AoA effect has been replicated in lexical decision tasks in English [4], Dutch [5]and Persian [6], in a semantic decision task in German [7], in free word recall tasks in Russian [8], in recognition memory tasks in English [9,10], as well as in picture naming and word recognition in both bilingual [11,12] and monolingual children [13].

The AoA effect has recently been also found in new tasks such as intentional and incidental forgetting [14], elicitation of tip-of-the-tongue experiences [15], as well as eye movement in reading [16]. In particular, early acquired words are more resistant to being forgotten, even if participants are instructed to forget the list of words [14]. Consequently, early words appear less often in tip-of-the-tongue phenomena, when a participant knows a word but cannot produce it [15]. Early words are also read faster, and lead to shorter fixation durations during novel reading [16]. Similarly to single words, an AoA effect has recently been shown in processing of multiword phrases: early acquired multiword phrases are processed faster [17].

AoA ratings are negatively correlated with word frequency, i.e. the higher the word’s frequency, the earlier it is acquired [18–27]. However, previous research has shown that the effects of AoA and word frequency are not identical and AoA effects may also be registered when word frequency is controlled in such tasks as picture naming or lexical decision [28–36]. On the other hand, a words’ AoA may depend on its frequency trajectory, i.e. the distribution of experiences of the word in time [23,37–39]. For that reason, authors suggested the use of frequency trajectory instead of AoA estimations to examine learning effects in lexical processing [38]. Nevertheless, application of either word frequency or frequency trajectory in cross-linguistic studies may be impossible, as finding comparable sources of word frequency in more than two languages may be challenging, and for many languages we still lack frequency data. In general, frequency data may be obtained in a language for each single word, when big enough corpus is available. This is not the case of subjective AoA ratings, which may be collected only for a limited set of words [40], even if the set of words is relatively big up to several thousand words [22,40–49]. However, in contrast to frequency data, AoA ratings may be collected in the same manner across a wide range of languages and cultures [1]. The difficulty with comparability of available sources of word frequency across languages and the independence of AoA and frequency effects, bring out the need for a cross-linguistic parallel database of AoA ratings.

### Order of word learning across languages

Although it is typically assumed that pace of vocabulary learning is similar across languages (despite acknowledging significant individual differences among children [50]), this assumption has not been studied systematically across a wide range of various languages [13].The pioneering research on modelling the order and pace of early word acquisition across seven languages is currently conducted with the use of the MacArthur-Bates Child Development Inventories (CDI) database [51]. First reports from this study suggested that the order of early acquisition of vocabulary may be predicted by the use of similar variables across languages, such as word frequency, concreteness and ‘babiness’ [51].

There are two basic methods of estimating at what age words are learned: an objective method based on wide studies of children acquiring the language [18,26,27,40,52–56], and a subjective one based on adults’ estimations of when they learned the words [1,19–21,41,44–46,49,53,57–96]. Both subjective and objective estimations of the age of word acquisition has been studied across several languages. The list of languages studied has been reviewed in detail [1] and includes such languages as Chinese, Dutch, English, French, German, Greek, Icelandic, Italian, Japanese, Norwegian, Persian, Portuguese, Russian, Spanish and Turkish. Recently new subjective estimations of AoA have been published for Arabic [97], German [47], Italian [98], Polish [48], Spanish [99,100] and Turkish [101] words. Though objective AoA ratings are, by definition, more valid than subjective estimations, they are much more difficult to obtain, as collection of objective AoA data involves studies of huge samples of children [1,18,26,27,40,55,56]. On the other hand, subjective AoA estimations are highly correlated to the objective measures [1,40,53,102,103].

Although either subjective or objective AoA data have been published for a wide list of languages, a direct comparison of the AoA estimations in two or more languages may be challenging, as usually the studies differ in terms of the list of items included in the study and in terms of the measurement scale used [1]. So far, the only study which employed the same method of data collection across 25 various languages from five language families was [1] and the current study broadens it with seven new languages. The previous study found moderate to high correlations of order of words between all language pairs under scrutiny. Existence of such correlations was earlier postulated [18,104], though the analysis was based on data collected in a various ways for the languages.

### The current study

Here we provide new AoA ratings for seven languages: American English (a Germanic language spoken in the US by 309 million people [105]), Czech (a West Slavic language spoken mostly in the Czech Republic with 10.4 millions of native speakers [105]), Gaelic (a Celtic language spoken as a minority language in Scotland by 57,400 people [105]), Lebanese Arabic (a dialect of Arabic spoken primarily in Lebanon by 94% of the Lebanese population though there are no precise estimations of its number [106]), Malay (a major language of the Austronesian family spoken by 19 million speakers in the world with 12.5 million speakers in Malaysia [105]), Persian (standard Iranian Persian spoken in Iran as the official language by 50 million people [105] in contrast to other variants of Persian spoken by 120 milion people worldwide [107]) and Western Armenian (one of the two standard forms of the Armenian language, spoken worldwide in the Armenian diaspora by 1.2 million people [105], outside the Republic of Armenia; in the current study all participating speakers of Western Armenian lived in Lebanon). For five languages included in the current paper (except for American English and Persian) no previous AoA data were available so far, which enhances the novelty of the current study.

We used a homogenous method of data collection in order to obtain ratings comparable across the seven languages studied in the current paper. Because we used the same method as in the previous study in 25 languages [1], the current paper results in a comparable database of AoA ratings in 32 languages (see S1 Table). In addition to providing the new database of AoA ratings, we asked the question of whether the previously found pattern of cross-linguistic similarities in the order of word acquisition occurs also among the languages not included in the previous research. By applying hierarchical clustering, we explored the patterns of similarities of AoA ratings across languages. Across the seven languages, we searched for predictors of AoA estimations by comparing the words’ AoA ratings with words’ length and frequency. Finally, in order to characterize the early vocabulary across languages, in each language we examined whether the first and the latest words differ in terms of length in syllables and phonemes, as well as in the number of compound words.

The database of AoA ratings across 32 languages which came from the original set of 25 languages [1] combined with the current study may be of particular use in cross-linguistic studies, where comparable data for several languages are needed. For instance, AoA collected in the same way across languages may be applied as predictor in studies of vocabulary acquisition [11–13] or multilingual studies of lexical decision [6].

## Method

### Participants

Research was approved by Ethics Committe of Faculty of Psychology, University of Warsaw. Participants were 173 adults, with a minimum of 21 per language (total range: 21 to 37, *M* = 26.2, *SD* = 5.6; see Table 1). Participants were 119 females (69%) and 54 males (31%), aged 18 to 68 (*M* = 26.2, *SD* = 10.7). The group was unbalanced in terms of gender, however the previous study on AoA across 25 languages reported no significant difference in the mean ratings between the women and men [1]. Participants were recruited via academic communication (university subject pool or lecturers informing students about the study), social media and through neighbourhood networks as well as chain-referral sampling. Data were collected in the US (Pennsylvania, for American English); Czech Republic (for Czech); UK (Scotland for Gaelic), Lebanon (for Lebanese and Western Armenian); Malaysia (for Malay) and Iran (for Persian). Participants took part in the study voluntarily, and in the case of American English and Czech received course credits. In the case of Gaelic, participants obtained vouchers for online shopping. All participants reported their gender, age, education level, occupation, country of residence, native language, number of spoken and used languages, and number of they have. In all languages participants additionally reported the age of their children, and in Gaelic some additional questions regarding the current and previous use of languages were asked. All participants provided AoA ratings for all words included in the survey.

**Table 1:** Characteristics of the participants included in the analysis per language.

Language	N	Age			Females	
		M	SD	Range	N	Percent
American English	23	19.00	1.13	18–22	12	52%
Czech	26	22.85	4.94	19–45	23	88%
Gaelic	21	41.57	16.20	20–68	8	38%
Lebanese Arabic	21	22.43	2.54	19–28	21	100%
Malay	37	21.97	2.39	19–27	27	73%
Persian	21	34.24	11.32	18–66	6	29%
Western Armenian	24	33.92	9.57	21–62	22	92%
TOTAL	173			18–68	119	69%

All participant demographic data as well as their responses are available from: https://osf.io/ucegs/ (DOI: DOI 10.17605/OSF.IO/UCEGS).

### Stimuli

The same sets of 158 nouns and 141 verbs (a total of 299 words) were used in each language. In each language, the lists of exact word forms were obtained in a naming study. However, the procedure of selection of exact word forms differed between the languages.

In all languages except Lebanese Arabic, i.e. American English, Czech, Gaelic, Malay, Persian and Western Armenian, the lists of items were obtained in separate naming studies which used the same set of pictures drawn for Cross-Linguistic Lexical Tasks (LITMUS-CLTs, [108]). In the naming studies, a minimum of 20 native speakers of the target languages provided labels for the pictures. The most frequently given labels for each of 299 pictures were then used as target items in the current AoA study. In several cases, the naming procedure brought no evident dominant name for a picture but several different names were provided with similar frequency. For these cases the additional words (mostly synonyms of the target words) were added to the list applied in the AoA procedure for a given language. Thus the exact number of items in each language was: 169 nouns + 148 verbs for American English, 172 nouns + 155 verbs for Czech, 178 nouns + 153 verbs for Gaelic, 162 nouns + 149 verbs for Malay, 169 nouns + 155 verbs for Persian, and 135 nouns + 112 verbs for Western Armenian. In order to enhance the comparability of the ratings across the languages, only the ratings for the dominant names (the one most frequent label for a picture in a given language) are reported in the current paper and used in the statistical analyses (a total of 299 words, the same as used in the previous study).

In the case of Lebanese Arabic, the items were selected in the same manner as in the 25 languages reported in the study by Łuniewska and colleagues [1], following a previous online picture naming study conducted in 34 languages, aimed at selecting target words for Cross-linguistic Lexical Tasks, LITMUS-CLTs [108].

Although the exact lists of word forms resulted from the picture-naming procedures, in the current study only words were used as stimuli, without any pictures. In each of the languages both the picture naming study (going beyond the purposes of the present paper) and the AoA study (described here) were preparatory studies for an ultimate goal of designing Cross-linguistic Lexical Tasks for these languages.

To access the relationship between AoA and word characteristics for each language, we used three measures: (1) length in phonemes, (2) length in letters (Table 2), and (3) word frequency. In case of word frequency, data were available for four of the languages only: American English [109], Czech [110], Malay [111] and Persian [96] (only noun frequency).

**Table 2:** Characteristics of the stimuli used in each language.

Language	Word length in phonemes			Word length in letters			Compound words	Word frequency		
	M	SD	Range	M	SD	Range		M	SD	Range
American English	4.56	1.50	1–12	5.59	1.78	3–14	6.1%	2881.57	5061.91	12–43695
Czech	5.51	1.60	2–14	5.56	1.60	2–14	7.8%	353.40	1464.34	1–23201
Gaelic	6.10	2.28	2–15	8.48	3.50	2–20	11.8%			
Lebanese Arabic	6.10	2.04	3–15	4.65	1.73	2–13	7.2%			
Malay	5.85	1.78	3–12	6.16	1.95	3–13	8.9%	7311.32	17890.55	7–165012
Persian	7.87	3.42	2–22	6.59	2.85	2–16	46.7%	305.74	800.67	1–6264
Western Armenian	5.28	1.86	2–13	5.29	1.90	2–15	14.9%			

### Procedure

The same instructions as used in [1] were applied in all languages but Gaelic. All materials (the website, instruction, examples etc.) were translated from English into each of the languages involved by native speakers who were also researchers (linguists or psycholinguists).

The procedure was available online via a website designed exclusively for the purposes of the study (www.words-psych.org). The website was made available in all 32 languages (new languages and languages studied previously by [1] therefore participants could use their native language exclusively while using the website. After entering the website, participants were instructed to download a file and open it in Microsoft Excel (or Open Office). The file contained four sheets. The first sheet presented basic information about the study and the instructions, and the second sheet contained questions on the demographics of the participants. The lists of nouns and verbs were presented on the third and fourth sheets, respectively. All the instructions, questions and words were presented in the mother-tongue of the participants, i.e. the language of assessment. The original spreadsheets used in the study are available from: https://osf.io/ucegs/ (DOI: DOI 10.17605/OSF.IO/UCEGS).

Participants were asked to estimate at what age they had learned the words presented in the two sheets. The exact form of the instruction was: “For each word please estimate the age (in years) at which you think you learned this word; that is, the age at which you would have understood that word if somebody had used it in front of you, even if you did not use, read or write it at the time”. The exact form of the question was: “When did you learn the word?”. Participants were asked to type a number from 1 (if they thought they had learned the word when they were one year old) to 18 (if they thought they had learned the word when they were 18 or older). They were encouraged to guess the age if they were not sure and not to spend too much time on any single word. If they did not know the word, they were asked to enter “X” in the box.

To ensure that the participants understood the instructions, we provided four examples of both nouns and verbs acquired early and later in life. The examples were presented in a table that looked similar to the one filled out by the participants. Explanatory comments were added to the table (e.g. “Someone estimates that s/he learned the word ‘to ask’ at the age of 3 years.”).

The words on both the noun and the verb list were presented in random order, generated individually for each participant during the file downloading. In the *Nouns* and *Verbs* sheets, below the list of words, a short thank-you note was presented together with a reminder of the other sheet (“Thank you for filling in the table for nouns. Have you filled the table for verbs as well?”). Each participant was given the full list of words. Task duration was about half an hour. After filling in the file, participants were asked to upload it via the website or send it as email attachment to the address reserved for the purposes of the study.

In Gaelic, very similar procedure was used. The only difference was that instead of a downloadable Excel file, an online Qualtrics survey was used. Gaelic participants answered the same question (translated to Gaelic) as speakers of other languages, and used the same scale to give their responses. The noun and verb parts were separated and Gaelic participants answered the whole set of questions. A Gaelic version of the spreadsheet is also available from : https://osf.io/ucegs/ (DOI: DOI 10.17605/OSF.IO/UCEGS).

### Statistical analyses

We first calculated average age of acquisition of each word in each language. Then for each participant we calculated Pearson’s correlation coefficients between the average AoA in his/her language and his/her estimations. Next, for each language we calculated the average value and standard deviation of the correlation coefficients. We aimed at excluding data from participants whose correlation coefficients would be lower than language average minus 2SD, though there were no such participants and thus data from all participants were included in further analyses.

For each word in each language we calculated the average age of acquisition. In order to check the reliability of the data, we computed split-half reliability coefficients in each language. To compute the coefficients, we randomly divided participants of each language into two subgroups and calculated the Pearson’s correlation coefficients for the average AoA ratings provided by the two groups.

To verify cross-linguistic patterns of word order, we computed Spearman’s rho correlations between average AoA in each of the seven languages reported in the current study and 25 languages reported in [1]. Then we ran a hierarchical clustering of variables, i.e. languages, with the use of R package ClustOfVar [112] in order to explore the patterns of similarities of AoA ratings across languages. In this package, distances between variables and clusters are based on correlation coefficients.

To examine the relationships between words’ AoA and their other characteristics in each language we calculated Spearman’s rho correlations between average AoA and word length in phonemes and letters, as well as frequency, if there were applicable data. Then we selected the earliest and the latest 30 words (up to 37 if there were more words of the same AoA) in each language and compared the length in phonemes and letters of these words with the use of a series of t-tests. Additionally, we compared the number of compound words between the earliest and the latest words with the use of a series of chi-square tests. The exact values of stimuli characteristics (word length in phonemes and letters, information about compound words and word frequency) are available as S2 Table.

## Results

### Reliability of the data

The split-half reliability coefficients were very high and ranged between .88 and .97 (S3 Table).

### Descriptive results

**Fig 1:**
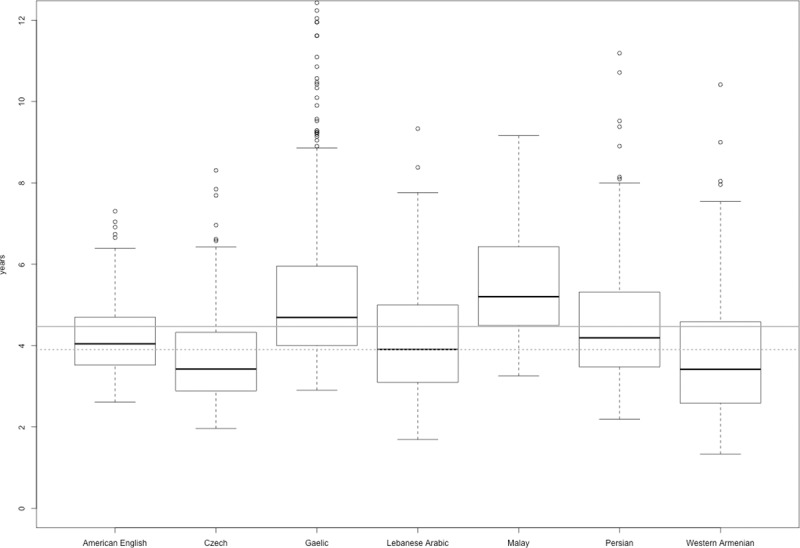
Means for AoA ratings across seven languages. The dots represent the words which are outliers. The solid line represents the mean for the seven languages (M = 4.50), the dotted line represents the mean for 25 languages (M = 3.90) studied in [1].

The average AoA ratings obtained for each of the seven languages are presented in the supplemental material. The vast majority of the words, namely 94% of the words, were assessed as known before the age of 7 years (Fig 1) and the remaining 6% of the words were estimated to be acquired between the age of 8 and 13 years.

### Cross-linguistic comparison

**Fig 2:**
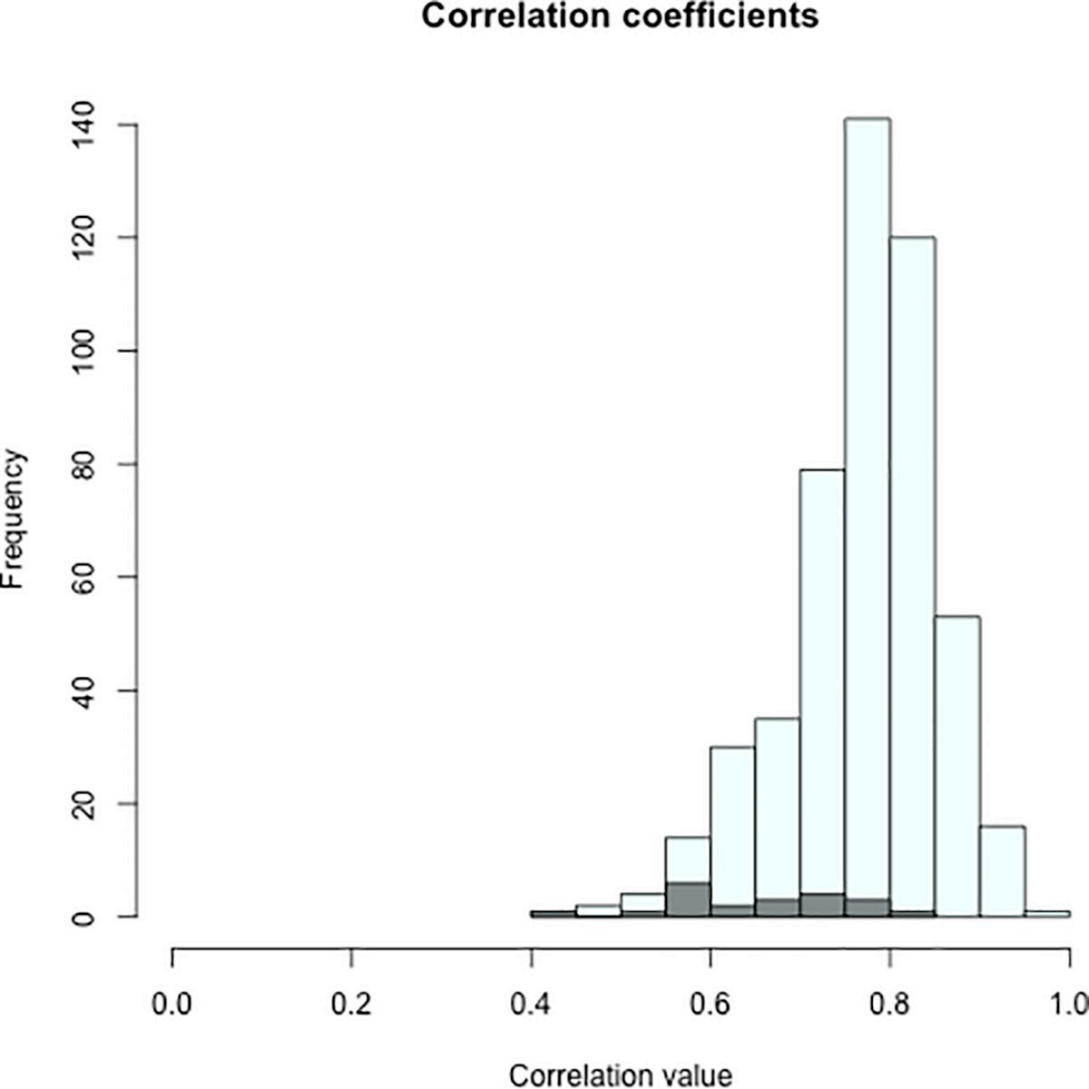
Histogram of correlation coefficients across 32 languages. The correlation values across the seven languages included in the current paper are marked with grey.

The ratings in the seven languages were moderately to strongly correlated with each other. Among the seven languages Spearman’s rho adjusted for split-half reliability varied from .45 for Gaelic-Malay pair to .85 for Western Armenian-Lebanese Arabic pair (see S3 Table and Fig 2). The ratings in the seven languages were also moderately to strongly correlated to previously collected ratings in [1]. For the seven languages contrasted to the 25 languages included in the previous study, Spearman’s rho adjusted for split-half reliability varied from .46 for Malay-Hungarian pair to .94 for Czech-Slovak pair, with mean and median for all languages equalled .77 and .78 (S3 Table and Fig 2).

Interestingly, while analysing the entire dataset (including the previously examined 25 languages and the added seven languages), we found a consistent pattern of correlations: (1) the languages close in terms of family had the highest correlation coefficients (American English vs. British English: .92, vs. South African English: .89; Czech vs. Slovak: .94, vs. Polish: .89, all being West Slavic languages; Lebanese vs. Maltese: .87, both being Semitic Languages; Gaelic vs. Irish: .88, both being Celtic languages); (2) Malay (a language from an Austronesian family not closely related to any other languages in the sample) had relatively low correlation coefficients (mean: .69), comparable with the mean correlation for Hungarian (mean: .68)–the language least similar to others included in the previous study [1], in terms of both origin and correlation coefficients obtained here; (3) Western Armenian (assessed in the Armenian minority in Lebanon) was most strongly related to Greek (a language said to be historically close to Armenian [113]): .87 and Lebanese (in the case of this study close not by language family but by territory): .84; (4) Persian had the strongest correlations with Turkish: .84, Maltese: .83, and Greek: .82.

**Fig 3:**
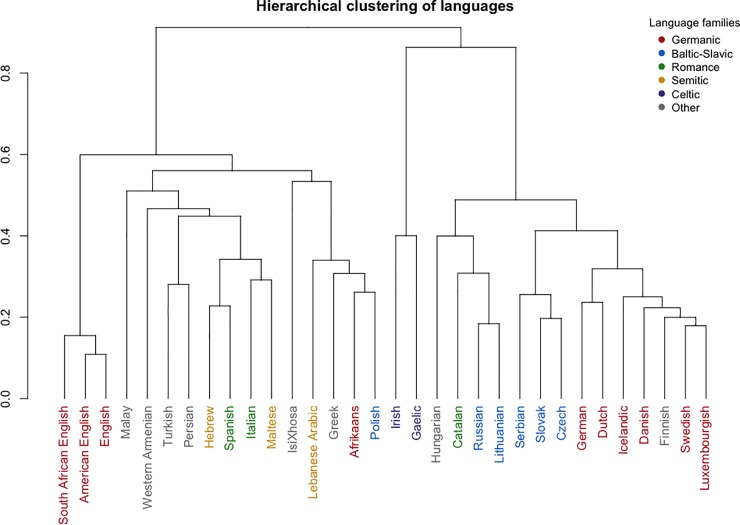
Similarity of AoA ratings across 32 languages. The language families are marked with colours. The distance on the Y-axis is equal to (1—Pearson’s correlation coefficient).

These patterns were mostly confirmed with the hierarchical clustering (Fig 3). In particular, in the hierarchical clustering we found that: (1) the three closest languages, forming the first cluster, were South African English, American English and British English, (2) most Germanic languages formed a joined cluster with Finnish, spoken in similar geopolitical settings, (3) Czech, Slovak and Serbian, all being Slavic languages, formed a joined cluster, (4) IsiXhosa and Malay, the single representatives of the Bantu and Austronesian languages, joined the clustering at the highest distance, i.e. were the least similar languages to all others. There are however some exceptions to this general pattern which should be noted: e.g. Polish is not clustered with other Slavic languages but with Afrikaans, a Germanic language, which in turn is not clustered with other Germanic languages.

### AoA and word characteristics

In all languages except Lebanese Arabic, both word length in phonemes and word length in letters were positively correlated to average AoA (Table 3). The significant correlation coefficients varied between low (.18 –.20 in Western Armenian) to moderately high (.47 –.50 in American English and .48 –.49 in Persian). Additionally, in all languages with existing frequency data we obtained moderately high negative correlations of AoA and word frequency (Table 3).

**Table 3:** Spearman rho correlation coefficients of words average AoA and word characteristics.

Language	Length in phonemes	Length in letters	Frequency
American English	.47***	.50***	-.54*** (n = 295)
Czech	.35***	.33***	-.45*** (n = 284)
Gaelic	.39***	.38***	
Lebanese Arabic	.16*	.14	
Malay	.36***	.35***	-.56*** (n = 291)
Persian	.49***	.48***	-.39*** (n = 106)
Western Armenian	.20**	.18**	

*** p < .001

** p < .005

* p < .01

Persian frequency data were available for nouns only

In all languages except Lebanese Arabic we found significant differences between the length of the earliest and the latest words in terms of both phonemes and letters (Table 4). The average difference between the earliest and the latest words’ length was 2.67 phonemes and 2.82 letters.

**Table 4:** Length and number of compound words in the earliest and latest words.

Language		Earliest Words	Latest words	
American English	Length in phonemes	3.29 (0.94)	5.97 (2.01)	t(61) = 6.75, p < .001***
	Length in letters	3.97 (0.91)	7.44 (2.33)	t(61) = 7.74, p < .001***
	Number of compound words	0/31	4/32	Chi^2^(1) = 4.14, p = .113
Czech	Length in phonemes	4.26 (1.15)	6.61 (1.43)	t(60) = 7.14, p < .001***
	Length in letters	4.35 (1.17)	6.58 (1.39)	t(60) = 6.83, p < .001***
	Number of compound words	1/31	3/31	Chi^2^(1) = 1.07, p = .612
Gaelic	Length in phonemes	4.35 (1.23)	7.27 (2.57)	t(65) = 6.10, p < .001***
	Length in letters	5.68 (2.35)	10.30 (3.80)	t(65) = 6.11, p < .001***
	Number of compound words	0/37	8/30	Chi^2^(1) = 11.21, p = .001**
Lebanese Arabic	Length in phonemes	5.42 (1.75)	6.74 (2.65)	t(60) = 2.32, p = .024
	Length in letters	4.19 (1.01)	5.42 (2.62)	t(60) = 2.39, p = .018
	Number of compound words	1/31	5/31	Chi^2^(1) = 2.95, p = .195
Malay	Length in phonemes	5.45 (1.68)	6.74 (1.85)	t(65) = 2.97, p = .004**
	Length in letters	5.76 (1.86)	7.15 (2.15)	t(65) = 2.84, p = .006*
	Number of compound words	2/33	4/34	Chi^2^(1) = 0.67, p = .673
Persian	Length in phonemes	5.03 (2.08)	11.40 (3.57)	t(58) = 8.45, p < .001***
	Length in letters	4.33 (1.71)	9.43 (2.60)	t(58) = 9.00, p < .001***
	Number of compound words	4/30	24/30	Chi^2^(1) = 26.79, p < .001***
Western Armenian	Length in phonemes	4.58 (1.31)	6.36 (2.55)	t(62) = 3.49, p = .001**
	Length in letters	4.65 (1.23)	6.36 (2.75)	t(62) = 3.20, p = .002**
	Number of compound words	4/31	9/33	Chi^2^(1) = 2.04, p = .217

*** p < .001

** p < .005

* p < .01

In two languages, Gaelic and Persian, we found a difference in compound word distribution between the earliest and the latest words (Table 4). In these two languages, there was significantly more compound words among the latest words than among the earliest ones.

## Discussion

We present a new database of subjective AoA ratings for 299 in seven languages from three language families which extends previously available ratings for 25 other languages [1]. Until now, there have been no published AoA ratings for five of these languages: Czech, Lebanese Arabic, Malay and Western Armenian; thus, the current study brings the first estimations of AoA in these languages. Although for American English the AoA ratings had already been available and for much bigger number of words, up to 30 thousand [44,46,89,91,93] and for Persian AoA ratings had been collected for 200 words [96], the current study provided uniform assessment of a consistent set of 299 words across a wide range of languages and hence together with the previously published ratings for 25 languages [1] forms a database of AoA ratings for 32 languages altogether. The database may be used not only as a source of control variables in cross-linguistic research, but also lay foundations for future analyses of factors affecting the pace and order of word acquisition across the languages. So far, cross-linguistic AoA data has been applied in studies of language acquisition [114,115], but existence of comparable AoA ratings across languages opens the way for a branch of cross-linguistic studies of lexical decision or predictors of vocabulary knowledge in both monolingual and multilingual speakers. The ratings may be used both for parallel research in different languages and for experiments run in both/all languages of a bilingual/multilingual sample.

The presented ratings have very high internal reliability (as indicated by values of split-half coefficients, see S3 Table). The stability of the reported age ranges across languages (see Fig 1) makes the list of words included in the study a possible source of target words in research on word learning in early childhood across many different languages. Additionally, the data validity in American English, Czech, Malay and Persian has been confirmed by moderately high negative correlations of the AoA estimations and word frequency (see Table 3). The obtained values of correlation coefficients were of a similar size as in previous research [18–26].

Similarly to [1], we found that the words included in the current study are reported to be learned relatively early in life, typically by the age of 7 years. The earliest words, i.e. the words with the lowest AoA in particular languages, were estimated to be acquired between the age of 1;4 (years; months; ‘dummy’ in Western Armenian) to 2;8 (‘ball’ in American English). Perhaps these values are overestimated and the words are in fact understood by younger children. This overestimation may partly result from the applied scale which started from ‘1 year’ instead of ‘0 years’ which could give lower estimations and from the one-year intervals used. Monthly intervals could give more precise estimations for the earliest words, but they would be inconvenient for estimating later acquired words (those estimated to be acquired above the age 3 years, up to the age of 11 years in the current sample). The other reason for the overestimation of the current ratings may lay in the exact question form: similarly to previous research [1,19,81] we asked participants to estimate when they had learned the words and this question may reveal higher estimations of AoA than a question “When do children speaking your language learn the word?” [1].

As we collected subjective AoA ratings, based on participants’ estimations rather than on data from studies of children acquiring a given language, the ratings may be biased in the same way across all languages. In fact, the subjective AoA ratings may illustrate participants’ intuitions about language acquisition more than real age of word learning. This idea seems to be partially supported by the found correlations between word length and AoA, present in six of seven studied languages (see Tables 3 and 4). It is plausible that adult speakers–who typically do not explicitly remember when they learned the earliest words–make their AoA estimations based on a word length cue. On the other hand, research shows that although subjective and objective AoA ratings do not result in the exactly the same values for each word, they are still highly correlated [84,102,103,116,117]. However, the reliability of the subjective AoA ratings may depend on raters’ experience with preschool children [118], as practitioners who work with children are more accurate in estimating exact values of words’ AoA, and this was not controlled in our study.

We found that the order of early word acquisition, as reported by adult native speakers, is similar in the seven languages included in the current study (Spearman’s rho coefficients adjusted for split-half reliability ranged between .49 and .84, see Table 2) and between the seven languages and the 25 languages analysed previously in [1] (Spearman’s rho coefficients adjusted for split-half reliability ranged between .48 and .94, see Table 2). The order of estimated word acquisition was similar across the languages, despite the fact that the additional languages included in the current paper represent three language families and data were collected in four continents (see Fig 2 and S3 Table). As such, the findings suggest a relatively universal order of word acquisition across a variety of languages and cultural settings. The correlations were particularly strong among close languages, such as variants of English, Germanic languages or Slavic languages, which was confirmed by hierarchical clustering (see Fig 3).

However, there are several other possible reasons for the correlations found across the languages. The present study is based on a preselected set of 299 words with rather specific properties: The referents of both nouns and verbs had to be easy to depict, as all the words have accompanying pictures in the CLT picture base [108]. The words were also chosen so that they could be expected to be known by children in the preschool age, as the words constituted a base of potential target words for vocabulary tests designed for this age. It is not clear to what extent the specific characteristics of the word list affect the correlation between AoA ratings and the actual age of word learning, as well as the correlations between AoA ratings across the languages. On one hand, restricting the set to early acquired words which are easy to depict might limit the range of possible responses and result in lower correlations between the measures. On the other hand, perhaps adults are much better at estimating children’s knowledge of words with specific and concrete meanings compared to more abstract words which were not examined here. It is possible that adult speakers of a language when providing estimations of AoA think of a context in which a child hears the assessed word. Imagining such context for concrete words may be easier than for abstract words: such contexts may involve direct contact with the object, e.g. playing with a ball or sucking a dummy. In the case of abstract words, conceptualising the situation in which a child hears the word may be more difficult and more diversified across participants and thus they may be less accurate in estimating words’ AoA. If so, this would lead to overestimated correlations between AoA ratings and the age of word learning and in AoA ratings across languages. It is a topic for further research whether the present results would be replicated with a broader and less semantically restricted set of words.

Finally, though the countries of data collection differ substantially in terms of culture, the background of the participants may be still quite similar, as they were recruited through academic networks. Perhaps application of another method of recruitment, e.g. invitation of random samples of speakers of the languages, would results in more variability in the obtained AoA ratings. However, so far research has not found any relationship between participants’ education level and the provided estimations of AoA [1,46].

## Supporting information

S1 Table Words’ AoA across 32 languages. (XLSX)

S2 Table Words’ characteristics (length in phonemes, length in letters, compound words, frequency) across languages. (XLSX)

S3 Table Matrix of correlations (Spearman's rank correlation coefficients adjusted for split-half reliabilities) of all languages with split-half reliabilities per language. All correlations significant: p < .001. Languages reported in the current study are printed in bold. Split-half reliabilities and coefficients of correlations for the language not reported in the current study are taken from [1] (Table 5).
(DOCX)
