# Supplementary material for: Age of acquisition of 299 words in seven languages: American English, Czech, Gaelic, Lebanese Arabic, Malay, Persian and Western Armenian
Source: PLoS One. 2019 Aug 8;14(8):e0220611. doi: 10.1371/journal.pone.0220611 (PMC6687123; doi:10.1371/journal.pone.0220611)
Supplement: S3 Table — All correlations significant: p < .001. Languages reported in the current study are printed in bold. Split-half reliabilities and coefficients of correlations for the language not reported in the current study are taken from [1] (Table 5). (DOCX) [file pone.0220611.s003.docx]

Supplementary Table 3. Matrix of correlations (Spearman's rank correlation coefficients adjusted for split-half reliabilities) of all languages with split-half reliabilities per language.

| Split-half reliability per language |  | Catalan | **Czech** | Danish | Dutch | **American English** | British English | South African English | Finnish | Gaelic | German | Greek | Hebrew | Hungarian | Icelandic | Irish | IsiXhosa | Italian | **Lebanese** | Lithuanian | Luxembourgish | **Malay** | Maltese | **Persian** | Polish | Russian | Serbian | Slovak | Spanish | Swedish | Turkish | Western Armenian |
| --- | --- | --- | --- | --- | --- | --- | --- | --- | --- | --- | --- | --- | --- | --- | --- | --- | --- | --- | --- | --- | --- | --- | --- | --- | --- | --- | --- | --- | --- | --- | --- | --- |
| .91 | Afrikaans | .84 | **.79** | .79 | .86 | **.81** | .83 | .89 | .80 | .64 | .85 | .81 | .78 | .72 | .75 | .80 | .87 | .78 | **.76** | .76 | .83 | **.73** | .82 | **.80** | .86 | .75 | .79 | .87 | .80 | .84 | .83 | **.73** |
| .91 | Catalan |  | **.75** | .76 | .76 | **.75** | .73 | .77 | .74 | .61 | .81 | .85 | .75 | .65 | .75 | .79 | .73 | .77 | **.76** | .75 | .82 | **.67** | .84 | **.75** | .86 | .74 | .75 | .81 | .84 | .82 | .79 | **.71** |
| **.91** | **Czech** |  |  | **.87** | **.85** | **.77** | **.83** | **.81** | **.79** | **.55** | **.85** | **.84** | **.78** | **.72** | **.75** | **.70** | **.74** | **.77** | **.72** | **.77** | **.83** | **.63** | **.81** | **.75** | **.89** | **.80** | **.88** | **.94** | **.79** | **.88** | **.80** | **.74** |
| .92 | Danish |  |  |  | .88 | **.80** | .86 | .85 | .83 | .70 | .83 | .87 | .81 | .72 | .82 | .78 | .76 | .79 | **.74** | .76 | .87 | **.60** | .86 | **.77** | .88 | .78 | .79 | .85 | .81 | .90 | .80 | **.73** |
| .92 | Dutch |  |  |  |  | **.79** | .85 | .83 | .83 | .66 | .89 | .84 | .79 | .72 | .82 | .75 | .76 | .80 | **.79** | .75 | .89 | **.62** | .84 | **.73** | .87 | .78 | .81 | .85 | .78 | .88 | .76 | **.74** |
| **.91** | **American English** |  |  |  |  |  | **.92** | **.89** | **.78** | **.66** | **.78** | **.79** | **.76** | **.58** | **.73** | **.74** | **.64** | **.78** | **.75** | **.67** | **.77** | **.58** | **.73** | **.70** | **.80** | **.76** | **.74** | **.77** | **.73** | **.78** | **.71** | **.69** |
| .99 | British English |  |  |  |  |  |  | .92 | .82 | .69 | .83 | .83 | .81 | .65 | .75 | .80 | .69 | .84 | **.77** | .70 | .84 | **.65** | .83 | **.73** | .85 | .80 | .80 | .84 | .81 | .84 | .76 | **.77** |
| .94 | South African English |  |  |  |  |  |  |  | .81 | .64 | .82 | .83 | .81 | .68 | .77 | .77 | .74 | .78 | **.78** | .74 | .83 | **.68** | .83 | **.75** | .86 | .83 | .79 | .84 | .80 | .85 | .79 | **.73** |
| .94 | Finnish |  |  |  |  |  |  |  |  | .64 | .86 | .81 | .78 | .70 | .82 | .76 | .75 | .77 | **.79** | .74 | .89 | **.66** | .85 | **.75** | .87 | .81 | .77 | .87 | .77 | .88 | .80 | **.74** |
| **.88** | **Gaelic** |  |  |  |  |  |  |  |  |  | **.56** | **.65** | **.55** | **.48** | **.70** | **.87** | **.61** | **.60** | **.56** | **.61** | **.68** | **.45** | **.61** | **.52** | **.61** | **.59** | **.52** | **.61** | **.55** | **.62** | **.61** | **.55** |
| .92 | German |  |  |  |  |  |  |  |  |  |  | .87 | .82 | .77 | .78 | .75 | .78 | .82 | **.79** | .76 | .90 | **.64** | .89 | **.76** | .89 | .81 | .85 | .88 | .82 | .87 | .82 | **.79** |
| .89 | Greek |  |  |  |  |  |  |  |  |  |  |  | .83 | .66 | .79 | .77 | .84 | .90 | **.84** | .76 | .85 | **.65** | .94 | **.82** | .90 | .79 | .84 | .90 | .85 | .82 | .86 | **.87** |
| .96 | Hebrew |  |  |  |  |  |  |  |  |  |  |  |  | .65 | .71 | .69 | .73 | .78 | **.79** | .70 | .80 | **.65** | .89 | **.78** | .84 | .78 | .75 | .81 | .84 | .81 | .79 | **.81** |
| .87 | Hungarian |  |  |  |  |  |  |  |  |  |  |  |  |  | .66 | .64 | .70 | .62 | **.57** | .69 | .73 | **.46** | .69 | **.64** | .72 | .71 | .69 | .78 | .69 | .71 | .68 | **.58** |
| .91 | Icelandic |  |  |  |  |  |  |  |  |  |  |  |  |  |  | .77 | .70 | .70 | **.74** | .72 | .85 | **.57** | .78 | **.71** | .79 | .77 | .73 | .83 | .71 | .83 | .75 | **.71** |
| .78 | Irish |  |  |  |  |  |  |  |  |  |  |  |  |  |  |  | .78 | .73 | **.71** | .76 | .83 | **.64** | .82 | **.72** | .79 | .69 | .71 | .76 | .75 | .75 | .78 | **.71** |
| .68 | IsiXhosa |  |  |  |  |  |  |  |  |  |  |  |  |  |  |  |  | .69 | **.76** | .68 | .78 | **.65** | .82 | **.71** | .78 | .71 | .74 | .76 | .75 | .77 | .80 | **.72** |
| .93 | Italian |  |  |  |  |  |  |  |  |  |  |  |  |  |  |  |  |  | **.80** | .65 | .83 | **.61** | .90 | **.76** | .87 | .74 | .79 | .83 | .81 | .77 | .76 | **.81** |
| **.94** | **Lebanese** |  |  |  |  |  |  |  |  |  |  |  |  |  |  |  |  |  |  | **.69** | **.82** | **.59** | **.87** | **.74** | **.78** | **.76** | **.76** | **.80** | **.76** | **.77** | **.77** | **.85** |
| .92 | Lithuanian |  |  |  |  |  |  |  |  |  |  |  |  |  |  |  |  |  |  |  | .78 | **.59** | .71 | **.66** | .80 | .84 | .75 | .83 | .73 | .80 | .76 | **.66** |
| .91 | Luxembourgish |  |  |  |  |  |  |  |  |  |  |  |  |  |  |  |  |  |  |  |  | **.64** | .91 | **.74** | .91 | .82 | .81 | .91 | .82 | .90 | .81 | **.76** |
| **.95** | **Malay** |  |  |  |  |  |  |  |  |  |  |  |  |  |  |  |  |  |  |  |  |  | .68 | **.63** | .71 | .60 | .61 | .68 | .69 | .67 | .64 | **.58** |
| .75 | Maltese |  |  |  |  |  |  |  |  |  |  |  |  |  |  |  |  |  |  |  |  |  |  | **.83** | .91 | .75 | .81 | .85 | .88 | .85 | .83 | **.91** |
| **.91** | **Persian** |  |  |  |  |  |  |  |  |  |  |  |  |  |  |  |  |  |  |  |  |  |  |  | **.82** | **.71** | **.75** | **.78** | **.79** | **.73** | **.84** | **.79** |
| .91 | Polish |  |  |  |  |  |  |  |  |  |  |  |  |  |  |  |  |  |  |  |  |  |  |  |  | .84 | .87 | .95 | .85 | .88 | .85 | **.81** |
| .95 | Russian |  |  |  |  |  |  |  |  |  |  |  |  |  |  |  |  |  |  |  |  |  |  |  |  |  | .78 | .88 | .76 | .84 | .78 | **.73** |
| .93 | Serbian |  |  |  |  |  |  |  |  |  |  |  |  |  |  |  |  |  |  |  |  |  |  |  |  |  |  | .90 | .80 | .84 | .75 | **.75** |
| .89 | Slovak |  |  |  |  |  |  |  |  |  |  |  |  |  |  |  |  |  |  |  |  |  |  |  |  |  |  |  | .83 | .91 | .83 | **.75** |
| .92 | Spanish |  |  |  |  |  |  |  |  |  |  |  |  |  |  |  |  |  |  |  |  |  |  |  |  |  |  |  |  | .81 | .82 | **.80** |
| .90 | Swedish |  |  |  |  |  |  |  |  |  |  |  |  |  |  |  |  |  |  |  |  |  |  |  |  |  |  |  |  |  | .79 | **.74** |
| .93 | Turkish |  |  |  |  |  |  |  |  |  |  |  |  |  |  |  |  |  |  |  |  |  |  |  |  |  |  |  |  |  |  | **.81** |
| **.97** | **Western Armenian** |  |  |  |  |  |  |  |  |  |  |  |  |  |  |  |  |  |  |  |  |  |  |  |  |  |  |  |  |  |  |  |

All correlations significant: p < .001. Languages reported in the current study are printed in bold. Split-half reliabilities and coefficients of correlations for the language not reported in the current study are taken from [1] (Table 5).
